# The Role of M3 Muscarinic Receptor Ligand-Induced Kinase Signaling in Colon Cancer Progression

**DOI:** 10.3390/cancers11030308

**Published:** 2019-03-05

**Authors:** Mazen Tolaymat, Shannon M. Larabee, Shien Hu, Guofeng Xie, Jean-Pierre Raufman

**Affiliations:** 1 Department of Medicine, University of Maryland School of Medicine, Baltimore, MD 21201, USA; mazentolaymat@umm.edu (M.T.); shu1@som.umaryland.edu (S.H.); 2 Department of Surgery, University of Maryland School of Medicine, Baltimore, MD 21201, USA; slarabee@som.umaryland.edu; 3 Department of Medicine, University of Maryland School of Medicine and VA Maryland Health Care System, Baltimore, MD 21201, USA; gxie@som.umaryland.edu

**Keywords:** muscarinic ligands, muscarinic receptors, colon cancer, bile acids, acetylcholine

## Abstract

Despite a reduction in incidence over the past decade, colon cancer remains the second most common cause of cancer death in the United States; recent demographics suggest this disease is now afflicting younger persons. M_3_ muscarinic receptor (M_3_R) mRNA and protein are over-expressed in colon cancer, and M_3_R can be activated by both traditional (e.g., acetylcholine) and non-traditional (e.g., bile acids) muscarinic ligands. In this review, we weigh the data supporting a prominent role for key protein kinases downstream of M_3_R activation in promoting colon cancer progression and dissemination. Specifically, we explore the roles that downstream activation of the mitogen activated protein kinase/extracellular signal-related kinase (MAPK/ERK), protein kinase C, p38 MAPK, and phosphatidylinositol 3-kinase/Akt (PI3K/Akt) pathways play in mediating colon cancer cell proliferation, survival, migration and invasion. We assess the impact of M_3_R-stimulated induction of selected matrix metalloproteinases germane to these hallmarks of colon cancer progression. In this context, we also critically review the reproducibility of findings derived from a variety of in vivo and in vitro colon cancer models, and their fidelity to human disease. Finally, we summarize the therapeutic potential of targeting various steps from ligand-M_3_R interaction to the activation of key downstream molecules.

## 1. Introduction

Colorectal carcinoma (CRC) is a leading cause of cancer mortality; approximately 1.4 million new cases were diagnosed worldwide in 2012 with 693,900 deaths in the same year [1]. In the United States, in 2018, approximately 140,000 cases were diagnosed with approximately 50,000 deaths [2]. Despite improving screening rates and the availability of novel treatments, CRC remains the second and third leading cause of cancer death in the United States for men and women, respectively. Furthermore, despite decreasing rates in those older than 55 years, the incidence of CRC among those aged 20 to 55 years appears to be increasing [3] with a concomitant rapid increase in the incidence of metastatic CRC in younger people [4,5]. This is especially worrisome as early-stage CRC is usually amenable to surgical resection, whereas metastatic disease responds poorly to chemoradiation and its prognosis remains poor with a 13.8% 5-year survival [6]. Thus, expanded efforts are necessary to understand the underlying mechanisms and develop effective therapies for advanced-stage CRC.

Cellular proliferation and escape from programmed cell death are hallmarks of neoplasia. Complex signaling pathways govern these processes, and consequently newer chemotherapeutic approaches are being developed to target these pathways. An example of such a promising target that resulted in new therapies over the past decade, is epidermal growth factor receptor (EGFR) signaling. EGFR, expressed broadly on normal human epithelial tissue, plays a prominent role in the progression of several cancers, including CRC. Monoclonal antibodies targeting EGFR ligand binding and small molecular inhibitors of EGFR kinase activity have provided modest benefit in treatment outcomes. Yet, mutations in EGFR or in downstream targets confer resistance to therapy [7], which has prevented substantial improvements in overall survival rates. In addition, response to therapy varies based on the anatomic location of the tumor, suggesting differences at the molecular level between right- and left-sided colon neoplasia impact treatment responses and outcomes [8,9]. Similarly, the M_3_ muscarinic receptor (M_3_R) has been identified as an integral component promoting the proliferation of CRC cells, and governing other aspects of CRC cell function required for cancer progression. Interplay between M_3_R and EGFR appears to amplify downstream signaling and the ensuing effects on cell function. Here, with an emphasis on the role of key kinases, we review the activation of M_3_R by traditional and non-traditional ligands, downstream signaling cascades requiring kinase activation, and the end-results of these actions on cancer progression. We also explore the potential for leveraging these advances in knowledge to develop novel therapeutic approaches to target these pathways. 

## 2. The Muscarinic Receptor in CRC

Muscarinic acetylcholine receptors represent a small family of G-protein coupled receptors (GPCR) with five subtypes; current nomenclature refers to these as M_1–5_R [10,11]. Activation of the muscarinic M_1_, M_3_, and M_5_ receptors (MR_odd_) invokes phospholipid metabolism and an increase in cellular calcium concentration, whereas activation of M_2_ and M_4_ receptors (MR_even_) results in decreased cellular levels of cyclic adenosine monophosphate (cAMP) following inhibition of adenylyl cyclase activity. These muscarinic receptor subtypes are expressed in a wide variety of tissues (Table 1) where they mediate many of the actions of acetylcholine (ACh), are central to neuronal signaling, and contribute to a wide array of pathological conditions. Specific muscarinic receptor subtypes have been investigated as an element of or therapeutic target for dementia and schizophrenia [12,13], cardiomyopathies [14], overactive smooth muscles in the urinary tract [15], obstructive pulmonary disease [16], and others. Germane to the present review, muscarinic receptor function has been implicated in the proliferation and survival of various cancer cells [17,18,19,20]; Spindel [21] and Shah [22] summarized the findings of studies using a multitude of tissue types. 

The gastrointestinal tract expresses several muscarinic receptor subtypes. The control of gastric acid secretion from parietal cells results from a complex interaction of autocrine stimulation from neighboring epithelial enteroendocrine cells and neural input to parietal cell M_3_ receptors from the vagus nerve and enteric nervous system [23]. Similarly, crosstalk between M_1_ and M_3_ subtype receptors on gastric chief cells stimulates the release of the proenzyme pepsinogen [24]. Smooth muscle tone in the GI tract is regulated by an interplay of the autonomic and enteric nervous systems; evidence suggests that M_1_ and M_3_ receptors play key roles in signaling [25]. Furthermore, the M_3_ receptor is expressed by normal colon tissues [26] and, as reviewed below, several studies reported over-expression of M_3_R in the majority of colon cancers.

Kopp et al. studied the binding affinity of subtype-specific muscarinic antagonists using HT-29 colon cancer cells [27]. Various antagonists were tested for their ability to block the formation of inositol phosphates after exposure to carbamylcholine (carbachol), a non-selective muscarinic receptor agonist. The results indicated that this cell line almost exclusively expresses M_3_ subtype muscarinic receptors. In the same cell line, Zhang et al. used X-ray microanalysis measurement of intracellular ion concentrations to confirm the M_3_R subtype was responsible for ACh-induced signaling [28]. Frucht et al. tested a series of specific antagonists, using 10 different colon cancer cells lines, and found evidence that only the M_3_R subtype was expressed by the six cell lines responsive to carbachol [29]. This was further confirmed using reverse transcriptase-PCR (RT-PCR) to confirm qualitatively the expression of only M_3_R messenger RNA (mRNA).

To validate these findings in tissues taken directly from patients, surgical specimens were analyzed for expression of M_3_R, encoded by *CHRM3* [30]. To control for person-to-person variation in expression, specimens were compared to normal colon epithelium controls from the same patient. Quantitative RT-PCR revealed over-expression of *CHRM3* in 10 of the 18 samples examined. In most samples, immunohistochemical analysis using an M_3_R-selective antibody revealed increased staining in carcinomas compared to adjacent normal tissue. Intriguingly, staining in normal epithelium was limited to the basolateral surface whereas in neoplastic tissue staining was detected in the cytoplasm and diffusely along the cell membrane, findings in line with the observation that neoplastic cells commonly lose polarity.

The work described above showed overexpression of M_3_R/*CHRM3* in the majority of cancer samples studied and, importantly, revealed that the distribution of expression was altered, shifting from the basolateral membrane in normal tissue to more diffusely along the cell surface in cancer. These findings suggest that access to M_3_R and the nature and source of M_3_R ligands in neoplastic cells may differ from that in normal tissues. As reviewed below, activation of M_3_R stimulates cell proliferation and invasion, and resistance to apoptosis, essential attributes for the growth and spread of cancer cells. However, focusing on receptor expression alone is likely to be insufficient to explain resulting effects; barring autoactivation of receptors, a change in functional outcomes requires increased ligand availability as well as accessibility. In this context, the production of ACh by cancers and the potential role of non-traditional muscarinic agonists were examined.

Although typically regarded as a neurotransmitter exclusively produced at synaptic junctions, compelling evidence has emerged that ACh may be produced by both normal and neoplastic non-neuronal cells. The presence of the enzymatic machinery required for the production and release of ACh is reported in the esophagus [31], heart [32], and pancreas [33], among other organs. In these studies of non-neoplastic tissues, non-neuronal cells were able to utilize ACh for a variety of physiologic signaling functions in both a paracrine and autocrine manner. In the gut, Takahashi et al. found evidence that the enzymes required for ACh production are expressed in mouse intestinal epithelium and confirmed the presence of ACh using liquid chromatography and mass spectrometry [34]. They confirmed their findings in an organoid model of the intestine, which importantly does not contain neuronal tissue. Again, utilizing agonists and antagonists of the muscarinic receptor, they found non-neuronal ACh is involved in growth and differentiation of the stem cells that are responsible for continually repopulating the gut epithelium.

The ability to produce ACh also appears to be present in colon neoplasia. Three of six colon cancer cell lines tested expressed choline acetyltransferase (ChAT) as shown by gene (mRNA) and protein (immunohistochemistry) analyses, and the presence of ACh in cell culture medium was confirmed using a sophisticated highly-sensitive liquid chromatography approach [35]. Staining of surgical specimens of colon cancer and normal tissue for ChAT also showed greatly increased expression of the enzyme in cancer tissue. Collectively, these findings suggest colon adenocarcinomas both upregulate expression of M_3_R and the machinery necessary for ACh production, feeding into a signaling mechanism that promotes cancer growth and invasiveness.

Nonetheless, ACh is not the only ligand able to activate M_3_R in the colon. The surprising observation that bile acids can bind and activate muscarinic receptors on gastric chief cells [36], led to investigation of their interaction with muscarinic receptors in other tissues. It was subsequently found that bile acids bind to Chinese hamster ovary (CHO) cells transfected with a variety of MR subtypes, thereby inducing the expected post-receptor intracellular metabolic changes [37]. Molecular modeling demonstrating similar structure and surface charge distribution between lithocholyltaurine (LCT), a representative bile acid, and ACh may account for this binding ability. In H508 human colon cancer cells, bile acids increased cell proliferation, an effect blocked by atropine (a non-selective muscarinic receptor antagonist) and inhibitors of epidermal growth factor receptors (EGFR), confirming that proliferative effects are mediated by interplay between two very different classes of receptors, G protein-coupled receptors and receptor tyrosine kinases [38]. Evidence suggests that concentrations of bile acids that promote cell proliferation are achieved in the human cecum; an immediate post-mortem analysis of cecal contents from 19 human subjects demonstrated that one-third contained bile acid concentrations shown to be pro-proliferative in vitro [39]. Notably, these subjects had died of unnatural causes unrelated to colonic disease or neoplasia, and it is not known whether colon bile acid concentrations differ in persons with colonic neoplasia, perhaps as a consequence of differences in the gut microbiome. These findings, along with the observation that cancer cells often lose polarity and display basolateral receptors on luminal (apical) surfaces, suggest that bile acids can play an important role in CRC progression. Furthermore, the ability of bile acids to induce neoplasia has also been demonstrated; mice fed a diet with addition of deoxycholic acid developed more aberrant crypt foci, a precursor malignant lesion [40] as well as frank tumors [41]. Moreover, intestine-selective carcinogen treatment of mice with impaired bile acid transport and increased spillage of endogenous bile acids into the colon resulted in augmented colon neoplasia compared to wild-type mice with normal fecal bile acid levels [42,43].

These findings may partially explain the known association between the consumption of a Western diet and the development of CRC. Persons living in developed countries commonly have different rates of various cancers compared to those in developing countries, a finding likely due to lifestyle, environmental, dietary differences, along with dissimilar exposures to microorganisms. CRC in particular has been linked to diets rich in animal protein or fat, or those low in fiber, although these tend to occur together and it is difficult to ascertain whether effects result from one or both factors [44,45,46]. High fat consumption is associated with increased fecal bile acid levels [47], a consequence of increased bile acid production. Increased intestinal bile acid levels may be a key link between dietary fat consumption and CRC.

## 3. Post-M_3_R Signaling and the Role of Kinases

As discussed above, M_3_R, a GPCR coupled to the G_q_ class of G proteins, typically acts through upregulation of phospholipase C generating diacyl glycerol (DAG) and inositol 1,4,5-trisphosphate (IP_3_) and increased levels of intracellular calcium. Increased levels of DAG and calcium activate protein kinase C (PKC) and alter gene transcription via a pathway involving the small family of mitogen-activated protein kinases (MAPK); a key member is extracellular signal-regulated kinase-1/2 (ERK1/2). EGFR has also been implicated as part of a second pathway that links M_3_R activation to changes in gene expression. Daub et al. reported ‘transactivation’ of EGFR as a mechanism underlying MAPK activation in the response of rat cells to several GPCR agonists [48]. Inhibition of EGFR activation by antagonists or non-functional mutations reduced MAPK activation and suppressed downstream gene expression. This crosstalk between signal transduction pathways was further elucidated in a subsequent study of the role of the heparin-binding EGF-like growth factor (HB-EGF) [49]. HB-EGF is a membrane-bound glycoprotein that, when released extracellularly, can bind and activate EGFR. HB-EGF can also be ‘hijacked’ as a receptor for diphtheria toxin and facilitates its internalization into the cell and subsequent toxic effects [50]. In the aforementioned study, Prenzel et al. demonstrated that extracellular signals linked MRs to EGFR. Cells that overexpressed M_1_R were cultured with cells that overexpressed the human EGFR. Neither cell line alone responded to carbachol (a non-selective muscarinic agonist), however when co-cultured and treated with carbachol, EGFR was autophosphorylated in cells expressing EGFR. Furthermore, treatment of the cells with CRM197, a non-toxic mutant of diphtheria toxin that binds and inhibits HB-EGF, completely inhibited GPCR-mediated EGFR phosphorylation but not direct EGF-induced phosphorylation. This suggests that GPCR transactivation of EGFR was likely solely dependent on HB-EGF. The authors further confirmed that HB-EGF is indeed released from its cell surface-bound precursor proHB-EGF through the action of a matrix metalloproteinase (MMP). This activity, and subsequent activation of EGFR, was prevented by an MMP inhibitor, batimastat.

More specific to M_3_R, downstream activation of MAPKs appears to be mediated by a combination of both post-M_3_R and -EGFR pathways. Activation of MAPKs was studied in human embryonic kidney cells that express M_3_R [51]. In these experiments, inhibition of PKC only partially decreased MAPK (ERK1/2) activation in response to carbachol. The remaining activity was prevented by an inhibitor of EGFR tyrosine kinase activity and by an inhibitor of Src, another tyrosine kinase commonly found in cancer cells as a proto-oncogene whose activation encourages cell survival. In colon cancer this relationship is likely maintained as human CRCs frequently over-express EGFR [52,53] and, as reviewed above, M_3_R is often similarly over-expressed. Transactivation of EGFR following M_3_R activation was demonstrated in H508 human colon cancer cells [54]. These cells express both M_3_R and EGFR and demonstrate ERK1/2 phosphorylation (activation) in response to treatment with either ACh or EGF. These actions were blocked by two different inhibitors of EGFR. Interestingly, inhibition of PKC did not change ERK1/2 phosphorylation in response to ACh, suggesting that in this cell line M_3_R signaling may be completely mediated by EGFR. A similar study utilizing bile acids as the ligand demonstrated that bile acid-induced proliferation of H508 cells was mediated by EGFR phosphorylation as the proliferative effect was abrogated by inhibition of either M_3_R or EGFR [38].

As mentioned above, EGFR is transactivated in response to activation of M_3_R by the binding of HB-EGF. To serve as an EGFR ligand, HB-EGF, produced as a cell membrane-bound pro-ligand, must be released by proteolysis. Prenzel et al. [49] suggested the protease activity was likely provided by a matrix metalloproteinase (MMP), a finding later confirmed in H508 colon cancer cells [55]. H508 cells were treated with deoxycholyltaurine, another representative bile acid, and cell proliferation was measured after application of various inhibitors. Cell proliferation was predictably decreased in response to EGFR kinase inhibition, by antibodies to the ligand-binding domain of EGFR, by neutralizing antibody to HB-EGF and by an inhibitor of its release. Utilizing specific MMP inhibitors, the likely culprit was narrowed down to MMP-1 or MMP-7. However, recombinant MMP-7, but not recombinant MMP-1, induced ERK1/2 phosphorylation, an effect blocked when EGFR or HB-EGF were inhibited or neutralized. Finally, targeting MMP-7 with antibody or small interfering RNA (siRNA) prevented deoxycholyltaurine-induced cell proliferation. The finding that a MMP contributes to neoplastic growth concurred with the literature with regards to MMP activity in cancer, where this family of enzymes is known to be involved in regulating cell signaling as well as invasion, metastasis, and angiogenesis among other processes [56]. More specifically, MMP-7 had already been found to be associated with invasion and metastatic potential in CRC [57] however this was previously attributed only to direct breakdown of basement membrane proteins.

There are several potential targets downstream of EGFR. One that was already mentioned is Src, the proto-oncogene whose inhibition was found to decrease MAPK phosphorylation in embryonic renal cells [51]. Src is a non-receptor protein tyrosine kinase that regulates multiple different pathways. Important to this review, Src is known to be overexpressed in CRC where its activity increases metastatic potential and may contribute to resistance to chemotherapy [58]. Furthermore, Src is also known to interact with EGFR and activated in response to EGF binding. Src may also increase the activity of EGFR through its kinase activity [59,60,61]. Src has been shown to activate pathways that involve the MAPK family as well as phosphatidylinositide-3 kinase (PI3K) [58]. Indeed, inhibition of Src decreased ACh- as well as EGF-induced phosphorylation of ERK1/2 in H508 colon cancer cells [54]. Thus, Src is a potential link between activation of EGFR and downstream activation of MAPK (ERK1/2) in CRC. In more general terms, activation of MAPKs is part of a cascade of kinases that includes members such as Ras, Raf, and MAPK/ERK kinase (MEK) [62]. In these schema, direct activators of the MAPK family are broadly termed MAPK kinases (MAPKK or MAP2K); activators of MAPKKs are referred to as MAPKK kinases or MAP3K.

Alternative to the EGFR-mediated pathway, another MAPK, p38 MAPK (also referred to as p38) was found to help mediate ACh-induced changes in transcription. In two colon carcinoma cell lines that were tested for expression of MMP-1 (see ‘Effects of M_3_R Activation’, paragraph 4), inhibition of the above ERK pathway did not completely abolish expression. It was found that ACh induces phosphorylation and activation of p38 through PKC resulting in MMP-1 expression [63] in an EGFR-independent fashion. Inhibition of both EGFR or ERK and p38 did completely prevent MMP-1 expression. Further experiments of inhibition of a single target suggested crosstalk between the two relevant pathways such as the previously mentioned PKC-mediated activation of ERK1/2.

In terms of substrates for MAPK/ERK, there are over 100 known targets, too numerous to discuss here (see review by Yoon and Seger for a more detailed review of this topic [62]). Germane to the present review, many of these targets located in the nucleus are likely transcription factors necessary for expression of genes controlling cell proliferation as well as differentiation and key processes. Yet other MAPK/ERK targets are cytosolic, possibly involving additional pathways that may be related or unrelated to gene transcription. For example, in H508 colon cancer cells, ACh treatment stimulates phosphorylation of p90 ribosomal s6 kinase (p90RSK), whose primary substrate is located in ribosomes and thus likely contributes to regulating translation [54].

**Figure 1:**
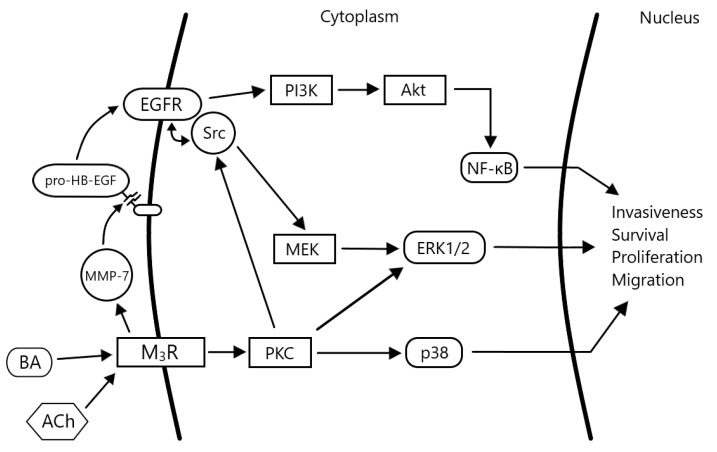
Complex signaling following activation of muscarinic receptors in colon cancer cells. Subtype 3 muscarinic receptors (M_3_R) are activated by bile acids (BA) or acetylcholine (ACh). The epidermal growth factor receptor (EGFR) is transactivated by heparin binding epidermal growth factor (HB-EGF), released from pro-HB-EGF by the actions of matrix metalloproteinase-7 (MMP-7), an enzyme whose expression and activation is also a result of M_3_R activation (a ‘feed-forward’ mechanism). Signaling proceeds downstream via the extracellular related kinase 1/2 (ERK1/2) and phosphatidylinositol-3-kinase (PI3K) pathways, thereby inducing changes in the transcription of genes that promote cancer progression (cell proliferation, survival, migration and invasion).

As alluded to above, PI3K is another potential downstream target of EGFR. PI3K is a family of kinases that respond to the activation of a variety of receptors, e.g., EGFR and insulin receptor [64]. Following its activation, PI3K phosphorylates phosphatidylinositol-4,5-bisphosphate (PIP_2_) to produce PIP_3_, a messenger that can bind several proteins. One such protein is Akt, itself a protein kinase with a litany of downstream targets including those that promote activation of the mammalian target of rapamycin (mTOR) and nuclear factor kappa B (NF-κB). Regulation and activity of both factors are impaired in many cancers. In a study using human HT-29 and H508 colon cancer cells, bile acid treatment induced Akt phosphorylation that was inhibited by EGFR kinase and PI3K inhibitors. The same inhibitors also attenuated bile acid-induced colon cancer cell proliferation. Additionally, bile acid treatment of these cell lines resulted in phosphorylation of glycogen synthase kinase 3 (GSK-3), Bcl-2-associated death promoter (or BAD, whose phosphorylation allows Bcl-2 to prevent initiation of apoptosis) and nuclear translocation of NF-κB [65]. It remains to be seen exactly which other actors downstream of M_3_R and EGFR are involved in signaling in colon cancer, but the appearance of several known proteins and enzymes in the studies above suggests they play key roles in mediating the various effects on cell proliferation and survival malignant cells after M_3_R activation (Figure 1).

## 4. Effects of M_3_R Activation

Several cellular functions appear to be governed by muscarinic receptor activation. Perhaps the most important is cell proliferation. In functional studies of muscarinic receptor subtype expression in 10 colon cancer cell lines, Frucht et al. focused on the effects of M_3_R [29]. Application of carbachol (the non-specific muscarinic agonist) to those cell lines demonstrated that M_3_R activation augmented cell proliferation by 25–133% depending on concentration of agonist used; this effect was blocked by various concentrations of *N*-methylscopolamine, a non-subtype selective muscarinic receptor antagonist. Cheng et al. showed the presence of ACh production machinery in colon carcinoma cell lines and used additional methods to validate their findings [35]. In H508 cells, antagonism of M_3_R as well as inhibition of choline transport reduced cell proliferation by ~40%. Conversely, antagonism of acetylcholinesterase (an enzyme catalyzing ACh hydrolysis) increased cell proliferation at least 2-fold.

These actions have also been demonstrated in vivo. Azoxymethane (AOM) is a known intestine-selective procarcinogen used to produce adenomas and adenocarcinomas in rodent models of colorectal neoplasia that mimic human colon neoplasia [66]. To demonstrate the effects of M_3_R activation on tumor growth in mice, wild-type and M_3_R-deficient mice were injected peritoneally with AOM or control solution [67]. Twenty weeks after the start of treatment, mice receiving control solution did not develop tumors, whereas most AOM-treated mice developed multiple colon tumors. M_3_R-deficient mice treated with AOM had a 40% reduction in number of tumors (mean 3.2 vs. 5.3 per mouse) and tumor volume was also reduced (by 60%). M_3_R-deficient mice were more likely to completely lack colon tumors and less likely to develop adenocarcinomas. Similar results were obtained with M_3_R deficiency in a mouse model of genetic CRC, so-called *Apc^Min/+^* mice [68]. Conversely, when mice were treated with AOM and bethanechol, a non-selective muscarinic agonist, colon tumor numbers and volume were augmented compared to mice treated with only AOM [69]. These results strongly implicate a role for M_3_R expression and activation in cell proliferation and tumor growth, and hint at the involvement of M_3_R earlier in the pathway to dysplasia and neoplasia.

These effects were also likely manifested in the rare case of an elderly man with unresectable pheochromocytoma [70]. Pheochromocytoma, a neuroendocrine tumor of the adrenal glands, classically secretes catecholamines which mediate elevated blood pressure and quickened heart rate. Pheochromocytomas have also been reported to produce ACh [71]. This man with a longstanding pheochromocytoma had a rectal adenoma removed endoscopically and, despite close surveillance, developed a rectal adenocarcinoma with unusual rapidity. The rectal carcinoma overexpressed M_3_R and the pheochromocytoma expressed ChAT suggesting that abundant Ach release from one tumor may have led to rapid growth of the other. As a parallel proof-of-concept, the addition of media from an ACh-producing pheochromocytoma cell culture to H508 cells increased cell proliferation substantially.

Muscarinic receptor activation in CRC may also stimulate cancer cell invasion and migration. MMP-7 catalyzed release of HB-EGF, results in transactivation of EGFR. MMP-1, another member of this metalloproteinase family, also appears to be induced by post-muscarinic receptor signaling. In a study of cell invasion, ACh and bile acids increased MMP-1 expression and the invasiveness of H508 and HT29 colon cancer cells in Matrigel-based and electrical cell impedance sensing assays [72]; these effects were blocked by pre-treatment with atropine or an anti-MMP-1 neutralizing antibody. Further studies of the inhibition of various steps along the M_3_R/EGFR/ERK1/2 pathway supported the conclusion that MMP-1 activation is likely a direct result of muscarinic receptor or EGFR activation [63]. In line with these findings in CRC, MMP-1 has been associated with invasive potential through its ability to break down extracellular matrix in other cancers. MMP-1 gene polymorphisms alter susceptibility to various neoplasms [73]. Finally, in a study of M_3_R expression in primary colon cancers, increased M_3_R expression was statistically associated with the presence of metastases [30]. However, immunohistochemical staining for M_3_R in lymph node and liver metastases was not increased compared to normal colon. This finding suggests that the increased invasiveness provided by muscarinic activation may be required to stimulate cell invasion and metastasis but not to maintain metastases once formed.

## 5. Potential for CRC Therapies

The above studies demonstrated that in mouse models of colon cancer, genetic manipulation of M_3_R expression or utilizing agonists or antagonists substantially affected both the development and growth of dysplastic and neoplastic lesions of the colon. The potential for therapeutic agents relying on this interaction should be explored, as there may be several advantages to targeting M_3_R. First, an oral agent with low to minimal systemic absorption could be employed to prevent or treat neoplasia limited to the colon while avoiding many of the adverse effects that would result from systemic anti-muscarinic receptor therapy. Second, an M_3_ receptor subtype-specific antagonist may further limit undesired effects. Third, as M_3_R is a surface receptor, medications targeting this receptor could evade drug efflux pumps, a common mechanism of resistance to chemotherapy. Conversely, decreased receptor production or availability in response to therapy may lessen effectiveness over time. Finally, staining for M_3_R in initial biopsy specimens may be a low-cost, rapid method to predict the presence of metastasis and responsiveness of patients to muscarinic antagonism, an example of patient-tailored precision oncology.

To our knowledge, currently no muscarinic receptor antagonists are used to treat cancer. However, several muscarinic receptor antagonists are available clinically and used for a variety of conditions. Notably, darifenacin, a muscarinic receptor antagonist approved by the U.S. Food and Drug Administration (FDA) to treat urinary incontinence, has considerable M_3_R specificity [74]. Several years of clinical experience with darifenacin suggest that M_3_R antagonists are safe and tolerated at approved doses—however, doses required for chemotherapeutic efficacy may differ. Further investigation of other subtype-specific antagonists should also be considered.

Other components of the signaling pathways highlighted in this review, including protein kinases, may provide other targets for cancer therapy. EGFR is already targeted in cancer; specifically monoclonal anti-EGFR antibodies (e.g., cetuximab) and EGFR kinase inhibitors (e.g., gefitinib) are FDA-approved to treat metastatic colorectal carcinoma [75]. Assays for EGFR expression are utilized to stratify patients for EGFR-based therapies as is analysis of mutations and expression of downstream mutations in RAS and BRAF that may predict treatment failure [76]. Another potential target is the MMP family of enzymes that governs both cancer cell invasiveness and transactivation of EGFR. MMP inhibitors successfully blocked EGFR activation in a prostate carcinoma cell line [49], however the lack of selectivity of currently available MMP inhibitors has impeded the translational potential of this class of agents. Clearly, M_3_R expression and activation governs colon cancer initiation and progression and the potential to use this to guide prognosis and therapy is worthy of further consideration.

## 6. Conclusions

The M_3_ muscarinic receptor subtype is over-expressed in colorectal carcinomas, where it plays an important role in key neoplastic processes including cell proliferation, survival, invasiveness, and metastatic spread. Although previously considered a product of only neuronal cells, acetylcholine, the prototypical ligand for this class of receptors, was shown to be produced by both normal and neoplastic non-neuronal cell types. In addition, although M_3_R is typically expressed on basolateral cell surfaces in healthy tissue, loss of cell polarity in neoplasia permits receptor expression on the luminal surface, thereby enabling bile acids in the fecal stream to bind and activate these receptors. This novel connection between bile acids and M_3_R may help to explain the association between high-fat Western diets and the risk of developing colorectal carcinoma. Once activated, M_3_R transactivates EGFR and both receptors initiate cascades of protein kinase activation leading to changes in gene transcription and protein expression that increase cell proliferation, stimulate tissue invasion and promote cancer cell dissemination. Thus, the role of M_3_R in CRC should be further explored, both to gain a better understanding of the mechanisms underlying neoplasia and to investigate their promise as therapeutic targets.

## Figures and Tables

**Table 1:** Muscarinic acetylcholine receptor subtypes. Primary signaling mechanisms and examples of tissue types expressing muscarinic receptor subtypes are listed.

Subtype	Gene	Mechanism of Signaling	Tissue Distribution
M_1_	*CHRM1*	Inositol phosphates	CNS ^1^, GI tract, lymphocytes
M_2_	*CHRM2*	Inhibition of adenylyl cyclase	Heart, smooth muscle (along stomach, bladder, airways, etc.), CNS
M_3_	*CHRM3*	Inositol phosphates	CNS, GI tract, smooth muscle
M_4_	*CHRM4*	Inhibition of adenylyl cyclase	CNS
M_5_	*CHRM5*	Inositol phosphates	CNS, esophageal smooth muscle, lymphocytes, salivary gland

^1^ CNS, central nervous system; GI, gastrointestinal.
